# Influence of confluent marine currents in an ecotonal region of the South-West Atlantic on the distribution of larval anisakids (Nematoda: Anisakidae)

**DOI:** 10.1186/s13071-018-3119-7

**Published:** 2018-11-08

**Authors:** Ana L Lanfranchi, Paola E Braicovich, Delfina M P Cantatore, Manuel M Irigoitia, Marisa D Farber, Verónica Taglioretti, Juan T Timi

**Affiliations:** 1 Laboratorio de Ictioparasitología, Instituto de Investigaciones Marinas y Costeras (IIMyC), Facultad de Ciencias Exactas y Naturales, Universidad Nacional de Mar del Plata - Consejo Nacional de Investigaciones Científicas y Técnicas (CONICET), (7600) Mar del Plata, 3350 Funes, Argentina; 2 Instituto de Biotecnología, Instituto Nacional de Tecnología Agropecuaria (INTA), Hurlingham, Buenos Aires, Argentina

**Keywords:** *Anisakis pegreffii*, *Anisakis berlandi*, *Anisakis typica*, *Zenopsis conchifer*, Zoogeographical indicators

## Abstract

**Background:**

In the marine environment, transitional zones between major water masses harbour high biodiversity, mostly due to their productivity and by containing representatives of species characteristic of adjacent communities. With the aim of assessing the value of larval *Anisakis* as zoogeographical indicators in a transitional zone between subtropical and sub-Antarctic marine currents, larvae obtained from *Zenopsis conchifer* were genetically identified. Larvae from *Pagrus pagrus* and *Merluccius hubbsi* from two adjacent zoogeographical provinces were also sequenced.

**Results:**

Four species were genetically identified in the whole sample, including *Anisakis typica*, *A. pegreffii*, *A. berlandi* and a probably new species related to *A. paggiae*. *Anisakis typica* and *A. pegreffii* were identified as indicators of tropical/subtropical and sub-Antarctic waters, respectively, and their presence evidenced the transitional conditions of the region. Multivariate analyses on prevalence and mean abundance of *Anisakis* spp. of 18 samples represented by 9 fish species caught south of 35°S determined that host trophic level and locality of capture were the main drivers of the distribution of parasites across zoogeographical units in the South-West Atlantic.

**Conclusions:**

Most samples followed a clear zoogeographical pattern, but the sample of *Z. conchifer*, composed mostly of *A. typica*, was an exception. This finding suggests that population parameters of *A. typica* and *A. pegreffii* could differ enough to be considered as a surrogates of the identity of larvae parasitizing a given host population and, therefore, a step forward the validation of the use of larval *Anisakis* as biological indicators for studies on host zoogeography.

**Electronic supplementary material:**

The online version of this article (10.1186/s13071-018-3119-7) contains supplementary material, which is available to authorized users.

## Background

Members of the genus *Anisakis* are known worldwide because of their implication in human health as the causative agents of anisakiosis, resulting from the ingestion of infective third-stage larvae in raw or undercooked marine fish products [1–4] and considered as one of the most significant emerging food-borne zoonoses [5]. Nevertheless, the biological relevance of anisakids in general goes beyond their epidemiological transcendence. Indeed, larval anisakids have been identified among the most suitable biological tags for stock discrimination because they have a lifespan or remain in an identifiable form in the host long enough to cover the timescale of such investigations [6–9]. However, a limitation to their effectiveness as markers is imposed by difficulties in their identification, since third-stage larvae of several species cannot be identified to species based on traditional morphological analyses, except to the level of the morphotypes of Berland [10], *Anisakis* Type I and Type II [11]. Some of these cryptic species often occur in sympatry and syntopy in fish hosts and the lack of taxonomic resolution can affect comparative studies. This limitation in specific reconnaissance for larval stages of *Anisakis* can, however, be solved with the application of molecular tools, which have recently proved to be of value when parasites are used as biological tags, especially for studies carried out at large geographical scales [12–15].

The success of larval *Anisakis* as tags to discriminate host populations at large spatial scales relies on the fact that members of this genus display species-specific distribution patterns within different climate zones and oceans which, in turn, are congruent with those of their respective final hosts [16]. For this reason, the species composition of these parasites in fish can reveal the transitional nature of ecotonal zones between zoogeographical marine regions or interface areas between masses of water, such that observed for hake and blue whiting between the cold Atlantic and the warm Mediterranean waters [12, 17].

In the Argentine Sea, larvae of *Anisakis* are commonly reported in fish hosts (see [9] and references therein) as *Anisakis* sp. or *A. simplex* (*s.l.*). The only published reports of genetically identified species of *Anisakis* are that of *Anisakis pegreffii* in *Merluccius hubbsi* [4] and the skates *Sympterygia bonapartii* and *Zearaja chilensis* [18], and that of a single specimen of *Anisakis berlandi* in *S. bonapartii* [18], highlighting the considerable uncertainty existing in the species composition of this genus in this region. Adult *Anisakis* have been also reported in several species of cetaceans in the Argentine Sea [19], all of them based on morphological identifications, and most reported as *Anisakis* sp. or *A. simplex* (*s.l.*), although Berón-Vera et al. [20] also reported *A. physeteris*. A similar situation occurs in Brazilian waters, where some species have been morphologically identified in several species of marine cetaceans [21]. However, recent papers, based on genetic identification of larvae and adults, have recorded a higher diversity, *A. typica* being the most abundant and widely reported species of the genus, occurring in both cetaceans and fish hosts along Brazilian coasts [22, 23].

A promissory couple of species to evaluate the relative influence of confluent marine currents in the South-West Atlantic is represented by *A. typica* and *A. pegreffii*. According to data based on genetic identification, the former occurs in warmer temperate and tropical waters between 35°N and 30°S, whereas the distribution range of the later in the Southern Hemisphere extends in temperate to colder regions from 30°S to 60°S [4, 24]. However, in the South-West Atlantic, the border between the distributions of both species could be displaced to higher latitudes due to the influence of the Brazil Current, which flows southwards carrying subtropical waters to collide with the northward flowing Malvinas Current, composed of sub-Antarctic waters, on the continental slope around 38°S in the Argentine-Uruguayan Common Fishing Zone [25]. In the South-West Atlantic, cetacean species distribute differentially along a latitudinal-temperature gradient [26–28], and a contribution of different *Anisakis* species typical for warmer and colder regions should be expected in the confluence region. In a recent paper, Lanfranchi et al. [29] evaluated the utility of parasites as indicators of marine ecotones by analyzing data on the assemblages of long-lived larval parasites of *Zenopsis conchifer* inhabiting deep waters in the region of convergence between the Brazil and Malvinas currents, the southernmost limit of its distribution in the South American Atlantic. The ecology of *Z. conchifer* is little known; however, there is no evidence of migratory movements in the South-West Atlantic, except a shift towards deeper waters as fish grow [30]. Indeed, this fish is considered as a poor swimmer with restricted mobility [31, 32] and consequently constitutes a suitable model to evaluate the presence of infective stages of anisakids in their habitat, by acting as a passive sampler of the available larvae in their prey.

Lanfranchi et al. [29] included data on other host species recognized as harbouring parasite assemblages representative of neighbouring zoogeographical regions, characterized by these masses of water [9, 33, 34]. These waters, with subtropical and sub-Antarctic origins, affected the structure of parasite communities in the ecotone by acting as sources of infective stages of helminth species (acanthocephalans, nematodes, cestodes) typical of adjacent zoogeographical units, which were considered as reliable indicators to define such transitional regions. Among suitable markers, Lanfranchi et al. [29] reported larval *Anisakis* in *Z. conchifer*, identifying most of them as *A. simplex* (*s.l.*) based on morphology. These parasites were found at a prevalence of 77.3%, unexpectedly higher than the prevalences reported in more coastal fishes at similar latitudes. In the present paper, a genetic identification of a subsample of larval *Anisakis* from the same samples of *Z. conchifer* were carried out to assess the relative influence of sub-Antarctic and subtropical waters on the specific composition of this genus. Indeed, this region contained the distributional range of *A. pegreffii*; their occurrence in sympatry with *A. typica* should confirm the influence of the Brazil Current, as postulated by Lanfranchi et al. [29]. In fact, the only *Anisakis* larva found in a sample of ten *Z. conchifer* from Rio de Janeiro, Brazil was genetically identified as *A. typica* [35].

Prevalence and mean abundance of larval *Anisakis* in the Argentine Sea, presumed to be mostly *A. pegreffii*, follow a latitudinal pattern increasing southwards irrespective of the host species harbouring them [9, 18]. The locality of capture of *Z. conchifer* in the ecotonal region also provided the opportunity for evaluating whether population attributes of larval anisakids follow a general distribution pattern across fish species with a similar trophic level in the region, or if they depart from it. Such a departure could be considered not only as evidence of the presence of different species of *Anisakis* with their own distribution patterns, but also indicative of the influence of warmer waters on the distribution of *Anisakis* spp. in the subtropical-sub-Antarctic convergence region.

The aim of this paper is, therefore, threefold: (i) to unequivocally identify larval *Anisakis* in the Argentine Sea based on molecular techniques; (ii) to assess their value as zoogeographical indicators in an ecotonal zone; and (iii) to determine the possible drivers of the distribution of *Anisakis* spp. across fish species of similar trophic levels in the Argentine Sea. Our results will also contribute to the knowledge and inventory of this genus in the World Ocean, filling a gap on the extant knowledge on the distribution and global diversity of *Anisakis*.

## Methods

### Fish sampling and parasite inventories

**Fig. 1:**
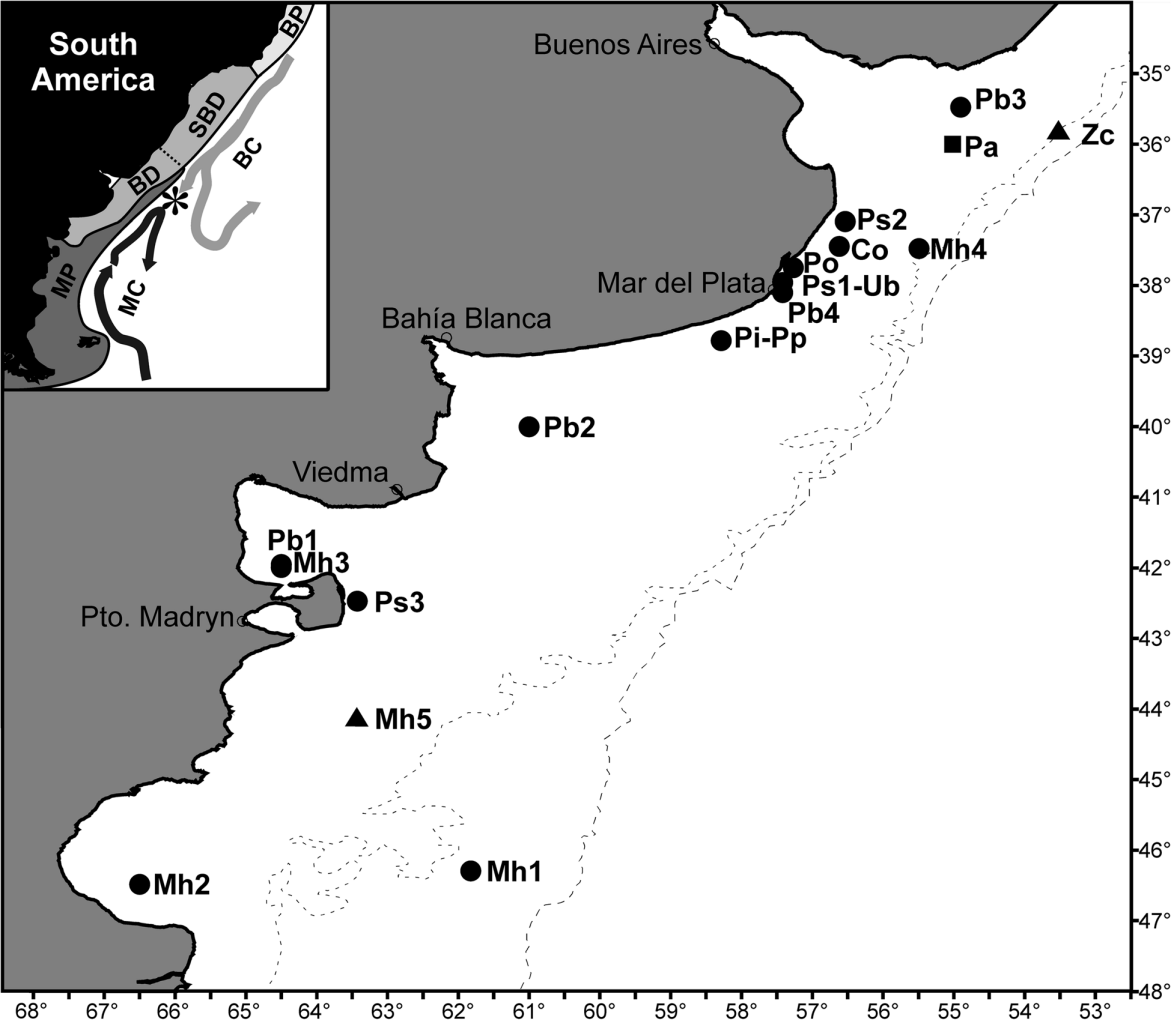
Map showing the sampling localities, the zoogeographical regions and the main marine currents. Circles, samples of fish species included in quantitative analyses (sample codes as in Table 1); square, sample of *Pagrus pagrus* (Pa) included in genetic analyses; triangles, samples of *Zenopsis conchifer* (Zc) and *Merluccius hubbsi* (Mh5) included in both genetic and quantitative analyses. *Abbreviations*: BP, Brazilian Province; SBD, South Brazilian district of Argentine Province; BD, Bonaerensean district of Argentine Province; MP, Magellanic Province; BC, Brazil Current; MC, Malvinas Current. Asterisk (*), ecotone region (convergence zone)

A total of 46 specimens of *Z. conchifer* were examined for larval *Anisakis.* Fish were caught by trawl during a research cruise at the Argentine-Uruguayan Common Fishing Zone (35°32'–35°35'S, 53°06'–53°25'W) at depths between 94 and 117 m, in October 2011 (Fig. 1). Data of most of these fish correspond to a previously published paper [29]. Fish were either kept fresh or deep-frozen in plastic bags at -18 °C until examination. Females (*n* = 31) measured on average 28.4 cm (range 16.5–46.5 cm), males (*n* = 15) measured on average 24.5 cm (range 17.0–35.0 cm). After defrosting, larval *Anisakis* were recovered from the mesenteries, body cavity and liver after examination under a stereoscopic microscope. Prevalence and mean abundance were calculated following Bush et al. [36].

### Genetic identification

The identification at the species level was obtained by direct sequences analysis of mitochondrial (mtDNA *cox*2) and nuclear (nDNA EF1 α-1) genes.

A subsample of 19 Type I larvae of *Anisakis* spp. (equivalent to 18.6% of the total collected) and a unique Type II larva, both from *Z. conchifer*, along with 16 preserved Type I larvae from another two fish hosts caught during previous studies (Fig. 1), all from the Magellanic zoogeographical province (9 worms from *Merluccius hubbsi*; 44°4'S, 63°29'W; January 2009), and Argentine zoogeographical province (7 worms from *Pagrus pagrus*; 36°S, 55°W; March 2016), were identified to the species level by genetic analyses.

DNA extraction was carried out using whole specimens with the DNeasy Blood and Tissue® Kit (Qiagen, Hilden, Germany) according to the manufacturer’s instructions. The mtDNA *cox*2 gene was amplified using the primers 210R (5'-CAC CAA CTC TTA AAA TTA TC-3') and 211F (5'-TTT TCT AGT TAT ATA GAT TGR TTY AT-3') [37]. Additionally, to confirm their identity, the nDNA EF1 α-1 was amplified in larvae of *A. pegreffii* selected among those identified by the mtDNA *cox*2 gene using the primers EF-F (5'-TCC TCA AGC GTT GTT ATC TGT T-3') and EF-R (5'-AGT TTT GCC ACT AGC GGT TCC-3') [38]. All PCR reactions were set up in a 25 μl reaction volume using 12.5 μl of HotStarTaq Master Mix (Qiagen), 0.5 μM of each primer and 5 μl of DNA (≥ 10 ng) as a template. The PCR was carried out using the following conditions: 94 °C for 15 min (initial heat activation) followed by 35 cycles at 94 °C for 30 s (denaturation), 50 °C for 100 s (annealing) and 72 °C for 1 min (extension), followed by a final extension step at 72 °C for 10 min. Each PCR product was purified using QIAquick spin columns (QIAquick Gel Extraction Kit, Qiagen). The fragments were sequenced for both DNA strands using the PCR primers. Sequencing was performed using Big Dye Terminator v.3.1 and 3130xl Genetic analyzer (Applied Biosystems, Foster City, CA, USA) at the Genomic Unit, IB-INTA.

Sequences were edited manually in Proseq v.3.5 [39] and deposited in the GenBank database. Generated sequences at the mtDNA *cox*2 were compared against the NCBI database using the BLAST algorithm [40]. The same sequences were aligned by ClustalW [41] implemented in the MEGA 7.0 software package [42] and compared with subsets of ten sequences available in GenBank of each known species of *Anisakis*, except for *A. physeteris* and *A*. cf. *paggiae*, of which only four and one sequences, respectively, were available for comparison. In the case of *A. paggiae*, those sequences deposited by Quiazon et al. [43, 44], considered as *A. paggiae*-related by the authors, were excluded due to specific uncertainty. The interspecific and intraspecific genetic distance between currently described *Anisakis* species and sequences obtained in the present study were calculated using a Kimura-2-Parameters (K2P) model in MEGA. The sequences obtained at the EF1 α-1 gene (409 bp fragment) of the nDNA for the specimens of *A. pegreffii* were compared at the diagnostic positions 186 and 286 as previously detailed in [38].

### Distribution of *Anisakis* spp. across fish species in the Argentine Sea

Data on prevalence and mean abundance of larval *Anisakis* in other fish species of the region were obtained from previous publications by the research team during the last 20 years (Table 1). Since host trophic level has been recognized as an important determinant of the abundance of long-lived parasites [45], and because the interest of the analyses relies on the geographical distribution of *Anisakis* spp., only those fish species with a high trophic level (> 3.5, obtained from Froese & Pauly [46]) and ichthyophagous diet [47] were retained (Table 1). In this sense, by having similar diets and due to the non-specificity of larval *Anisakis* in previous paratenic fish hosts, all host species are expected to be exposed to the same pool of infective stages, diminishing the effect of their trophic level, and therefore allowing other variables to arise as determinants of parasite burdens.

**Table 1:** Composition of samples used for comparative analyses on the distribution of larval *Anisakis* in the Argentine Sea, including number of examined hosts (*n*), latitude S (Lat) and longitude W (Long) of capture, trophic level (TL) and mean total length (MTL) of hosts, and prevalence (P in %) and mean abundance (MA) of parasites

Host species (sample code)	*n*	Lat S^a^	Long W^a^	Region	Year	TL	MTL (cm)	P (%)	MA	Reference
*Zenopsis conchifer* (Zc)	46	35.55	53.25	Ecotone	2011	4.17	27.45	78.3	2.17	Present study
*Conger orbignianus* (Co)	50	37.50	56.65	Bonaerensean	2010	3.72	80.69	8.0	0.12	[50]
*Merluccius hubbsi* (Mh1)	115	46.33	61.83	Magellanic	1999	4.23	39.91	89.6	52.60	[79]
*Merluccius hubbsi* (Mh2)	80	46.50	66.50	Magellanic	1998	4.23	40.36	97.5	16.60	[79]
*Merluccius hubbsi* (Mh3)	83	42.00	64.50	Ecotone	1999	4.23	38.68	100	17.00	[79]
*Merluccius hubbsi* (Mh4)	42	37.50	55.50	Ecotone	2009	4.23	44.50	52.4	1.36	[29]
*Merluccius hubbsi* (Mh5)	50	44.07	63.48	Magellanic	2009	4.23	41.80	98.2	46.96	[29]
*Paralichthys isosceles* (Pi)	51	38.87	58.17	Bonaerensean	2009	4	27.95	0	0	[80]
*Paralichthys patagonicus* (Pp)	51	38.87	58.17	Bonaerensean	2010	3.9	35.20	9.8	0.18	[80]
*Paralichthys orbignyanus* (Po)	44	37.74	57.42	Bonaerensean	2004-2008	3.8	51.00	3.8	0.03	[81]
*Percophis brasiliensis* (Pb1)	32	42.00	64.51	Ecotone	2006	4.3	52.90	53.1	8.70	[78]
*Percophis brasiliensis* (Pb2)	51	40.00	61.00	Bonaerensean	2006	4.3	50.00	98.0	16.50	[78]
*Percophis brasiliensis* (Pb3)	35	35.50	54.83	Bonaerensean	2006	4.3	49.40	11.4	0.20	[78]
*Percophis brasiliensis* (Pb4)	59	38.13	57.53	Bonaerensean	2006	4.3	52.20	33.9	0.60	[78]
*Pseudopercis semifasciata* (Ps1)	30	38.03	57.30	Bonaerensean	2007	3.9	71.20	30.0	0.67	[82]
*Pseudopercis semifasciata* (Ps2)	20	37.25	56.38	Bonaerensean	2007	3.9	67.50	0	0	[78]
*Pseudopercis semifasciata* (Ps3)	50	42.37	63.50	Magellanic	2007	3.9	67.20	72.00	4.48	[78]
*Urophycis brasiliensis* (Ub)	62	38.00	57.50	Bonaerensean	2012	3.8	36.60	0	0	[33]

^a^Central point of distribution when two or more trawls were made

Including data for *Z. conchifer*, a total of 18 samples corresponding to 9 fish species were analyzed. Each sample was assigned to a region following pre-established zoogeographical schemes ([48] and references therein); these regions were the Bonaerensean District of the Argentine Province and the Magellanic Province, both displaying characteristic parasite faunas [9]. Samples caught at transitional areas between these two regions [48], namely San Matías Gulf and the outer shelf of the Bonaerensean region, influenced by sub-Antarctic waters which at these latitudes flow northwards along the slope bordering shelf waters, were assigned to a third region, defined as ecotone.

To analyze the relative contribution of host/abiotic variables on parasite distribution, Euclidean distance matrices of both prevalence and mean abundance were analyzed by distance-based multiple linear regressions (DistLM) [49] with significance testing based on 9999 permutations. As host-related predictor variables, the host species and their trophic level and mean total length were included in the models due to their known influence on parasite burdens [45, 50, 51]. Abiotic predictor variables were latitude, longitude and year of capture. The central year of the study period (2006) was adopted as the date of the sample Po. Draftsman plots and correlation matrices were used to check for multicollinearity in the predictor variables. Latitude and longitude were highly correlated each other (*R* = 0.93), due to the northeast to southwest orientation of the Argentine continental shelf, but were combined in a single predictor (locality).

Models including all possible combinations of predictor variables were generated using the Best procedure within the DistLM routine. An information theoretic approach based on modified Akaike’s information criterion (AICc) was used to identify the best model; models with the lowest AICc were considered the most parsimonious [52]. The AICc was devised to handle situations where the number of samples (*n*) is small relative to the number (v) of predictor variables (*n*/v < 40) [49]. The difference (Δ_i_) between the AICc value of the best model and the AICc value for each of the other models was calculated; models with Δ_i_ between 0 and 2 are considered as having a substantial level of empirical support of the model being therefore as good as the best model [53]; however, as suggested by Richards [54], models with Δi up to 6 should not be discounted, thus all models with Δ_i_ ≤ 6 were retained. For each selected model, the Akaike weights (w_i_) were calculated following Burnham & Anderson [53] to identify and quantify the uncertainty in model selection and further used to estimate the relative importance of each predictor variable (predictor weight). For each predictor, the Akaike weights of all the models (with Δ_i_ < 6) that contained that predictor were summed and these values were interpreted as the relative importance of that predictor. Indeed, those predictors occurring consistently in the most likely models have a w_i_ close to 1, whereas variables that are absent from or are only present in poorly fitting models (high AICc values) have a w_i_ close to 0 [52]. Additionally, the relative strength of each candidate model was assessed by calculating the evidence ratio (ER), which provides a measure of how much more likely the best model is than alternative models [52].

To visualize possible geographical patterns in the prevalence of larval *Anisakis* across the 18 samples, non-metric multidimensional scaling (nMDS) was carried out using Euclidean distances. A hierarchical agglomerative clustering was applied to the component communities using complete linkage, and resemblance levels were overlaid on the nMDS plot [55].

All multivariate analyses were implemented in PERMANOVA+ for the PRIMER7 package [49, 55].

## Results

### General results

A total of 103 larval *Anisakis* were found parasitizing *Z. conchifer*. All of them were identified as Type I larva (prevalence: 78.3%; mean abundance: 2.2; range: 0–13), except for one specimen classified as Type II.

### Genetic identification

The identification through BLAST showed that sequences from the mtDNA *cox*2 gene of larvae *Anisakis* Type I exhibited a similarity of 99–100% with sequences for *A. typica* available on GenBank (*n* = 20; 16 from *Z. conchifer*, accession numbers MH443102-MH443117 and 4 from *P. pagrus*, accession numbers MH443118-MH443121); of 99% with those for *A. berlandi* (*n* = 3; 2 from *Z. conchifer*, accession numbers MH443122-MH443123 and 1 from *P. pagrus*, accession number MH443124); and of 99–100% with sequences for *A. pegreffii* (*n* = 12, 1 from *Z. conchifer*, accession number MH443127; 2 from *P. pagrus*, accession numbers MH443126-MH443123; and 9 from *M. hubbsi*, accession numbers MH443128-MH443136). The only larva *Anisakis* Type II from *Z. conchifer* (accession number MH443137) showed a similarity of 95% with the sequences for *A. paggiae* available on GenBank and of 99% with an undetermined species sequence, *A*. cf. *paggiae*.

Interspecific genetic distances (Table 2) ranged between 0.10–0.20, except for those corresponding to the *A. simplex* complex and the pair *Anisakis* sp.-*A*. cf. *paggiae*, which ranged between 0.05–0.06. The distances between the sequences from the present study and those from GenBank to which they showed the maximum similarity were all 0.01–0.02, the range of most of the observed intraspecific distance, thereby confirming their specific status.

**Table 2:** Averaged genetic distance calculated with the Kimura-2-parameter model at interspecific (below the diagonal) and intraspecific (diagonal, in italics) levels between specimens from the present study (denoted with *) and sequences deposited in GenBank

	A. sim	A. peg	A. peg*	A. ber	A. ber*	A. typ	A. typ*	A. zip	A. nas	A. phy	A. pag	A. cf. pag	A. sp.*	A. bre
A. sim	*0.02*	0.01	0.01	0.01	0.01	0.02	0.02	0.02	0.02	0.02	0.02	0.05	0.02	0.02
A. peg	0.05	*0.01*	0.003	0.01	0.01	0.02	0.02	0.01	0.02	0.02	0.02	0.04	0.02	0.02
A. peg*	0.05	**0.02**	*0.02*	0.01	0.01	0.02	0.02	0.01	0.02	0.02	0.02	0.04	0.02	0.02
A. ber	0.06	0.06	0.07	*0.01*	0.005	0.02	0.02	0.01	0.02	0.02	0.02	0.05	0.02	0.02
A. ber*	0.05	0.06	0.06	**0.02**	*0.02*	0.02	0.02	0.01	0.02	0.02	0.02	0.05	0.02	0.02
A. typ	0.14	0.14	0.14	0.14	0.14	*0.02*	0.003	0.02	0.02	0.02	0.02	0.06	0.02	0.02
A. typ*	0.14	0.14	0.13	0.14	0.14	**0.02**	*0.01*	0.02	0.02	0.02	0.02	0.06	0.02	0.02
A. zip	0.11	0.12	0.12	0.13	0.12	0.15	0.15	*0.02*	0.10	0.01	0.01	0.03	0.02	0.02
A. nas	0.14	0.13	0.13	0.14	0.13	0.13	0.13	0.10	*0.01*	0.02	0.02	0.04	0.02	0.02
A. phy	0.13	0.13	0.13	0.13	0.13	0.16	0.16	0.13	0.16	*0.01*	0.02	0.04	0.02	0.02
A. pag	0.13	0.12	0.12	0.14	0.14	0.17	0.17	0.12	0.14	0.13	*0.03*	0.02	0.01	0.01
A. cf. pag	0.16	0.15	0.15	0.17	0.17	0.20	0.21	0.12	0.16	0.16	0.06	–	0.01	0.04
A. sp.*	0.15	0.14	0.14	0.15	0.15	0.19	0.19	0.12	0.15	0.14	**0.06**	**0.01**	–	0.01
A. bre	0.16	0.16	0.16	0.17	0.17	0.20	0.20	0.14	0.18	0.11	0.13	0.13	0.12	*0.02*

*Abbreviations*: A. ber, *A. berlandi* (GenBank: KC809999-KC810001, JF423292-JF423297, MF353876); A. bre, *A. brevispiculata* (GenBank: KY421194, KP992462, EU560909, DQ116433, KJ786284, KJ786285, KC342900, KC342901, AB592803, AB592805); A. nas, *A. nascettii* (GenBank: FJ685642, GQ118164-GQ118169, GQ118171, GQ118173, JQ010980); A. phy, *A. physeteris* (GenBank: DQ116432, KU752202, KC479947, KC479948); A. sim, *A. simplex* (*s.l.*) (GenBank: KC810004, KC810003, KX158869, GQ338432, KT852475, KC479861, AB517570, JF423230, EU413959, MF358545); A. zip, *A. ziphidarum* (GenBank: KP992461, KT822146, DQ116430, KU752204, KU752205, KC821735, KC821737, KC821738; KF214804, KF214805); A, pag, *A. paggiae* (GenBank: KF693769, DQ116434, DQ116434, KJ786280, KJ786277, KJ786276, AB592809, AB592808, AB592810); A. cf. pag, *A. paggiae* related (see Di Azevedo et al. [56]) (GenBank: KF693770); A. sp., *Anisakis* sp.; A. peg, *A. pegreffii* (GenBank: EU933995, JQ341912, KR149283, KC480025, KC479888, KC479890, KC479993, KC809996, MF353877, MF353878); A. typ, *A. typica* (GenBank: KC821729, JQ859931, KP992467-KP992469, DQ116427, KF356646, KF032065, KF032063, KF701409)

Within group standard errors are given above the diagonal. Genetic distances between sequences of the present study and those from GenBank for the same species are in bold

In addition, the presence of a T at the position 186 and of a C at the position 286 of the partial sequences of the EF1 α-1 region of the nDNA obtained in 8 specimens, confirmed the identification of *A. pegreffii* (GenBank: numbers MH443138-MH443136).

### Distribution of larval *Anisakis* across fish species in the Argentine Sea

**Fig. 2:**
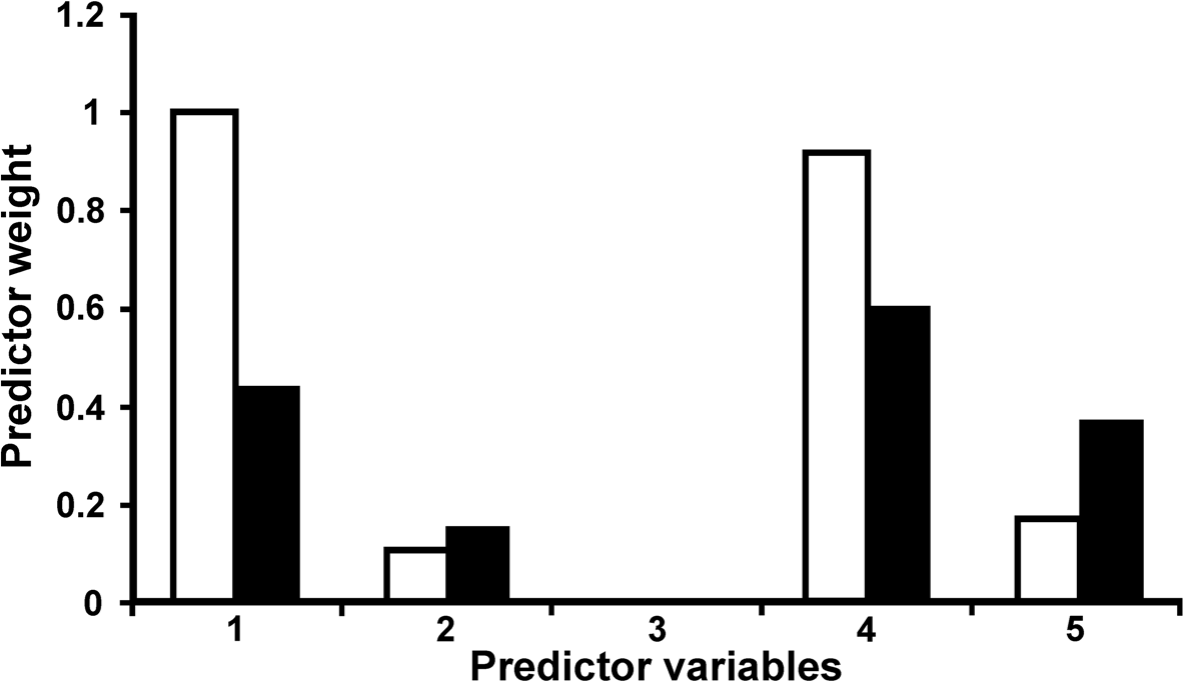
Predictor weights of variables included in models with Δ_i_ < 6 resulting of the DISTLM analyses on prevalence and mean abundance of *Anisakis simplex* (*s.l*.) in 18 samples corresponding to 9 fish species from the southern South-West Atlantic coasts. White bars, prevalence; black bars, mean abundance. Predictor variables: 1, trophic level; 2, mean host length; 3, host species; 4, locality; 5, year

The results of the DistLM on the prevalence data showed that the best model included host trophic level and locality as predictor variables (explaining 69.8% of the total variation of the data) (Table 3). The w_i_ indicated that it has 69.0% chance of being the best model and ER showed that it was nearly five times more likely to be the best approximating model than the subsequent one. Indeed, trophic level and locality were included in all and most models with Δ_i_ < 6, reaching a predictor weight of 1 and 0.9, respectively, indicating that both variables had the highest probabilities of being a component of the best model (Fig. 2). Regarding mean abundance, a higher number (10) of alternative models were obtained, the best one composed only by locality as a predictor variable (explaining 38% of the total variation of the data) (Table 3). The w_i_ indicated that the first model has a 22.8% chance of being the best one, a value very similar to that of the subsequent (composed by locality and trophic level). Evidence ratios showed that the first two models had similar chances of being the best one, but both were more than one and a half times more likely to be the best approximating models than the subsequent one. The predictor weights indicated that locality had the highest relative importance, followed by trophic level and year with considerably lower importance, whereas the mean host length had quite negligible relevance (Fig. 2). None of the models with Δ_i_ < 6 included host species as explanatory variable for the prevalence or mean abundance.

**Table 3:** Summary table of the results of the DISTLM analysis on prevalence and mean abundance of larval *Anisakis* in 18 samples corresponding to 9 fish species from the South-West Atlantic coasts. Results are ordered by the modified Akaike information criterion and only those models with Δ_i_ < 6 are included

Response variable	Model	AICc	*R* ^2^	Predictors^a^	Δ_i_	Wi	ER
Prevalence	P1	121.28	0.70	1, 4	0	0.6903	–
	P2	124.65	0.71	1, 4, 5	3.37	0.1280	5.39
	P3	125.15	0.70	1, 2, 4	3.87	0.0997	6.92
	P4	126.78	0.42	1	5.50	0.0441	15.64
	P5	127.09	0.5	1, 5	5.81	0.0378	18.26
Mean abundance	MA1	97.83	0.38	4	0	0.2278	–
	MA2	98.01	0.48	1, 4	0.18	0.2080	1.09
	MA3	98.57	0.24	5	0.74	0.1573	1.45
	MA4	99.56	0.20	1	1.73	0.0959	2.37
	MA5	99.98	0.30	1, 5	2.15	0.0778	2.93
	MA6	100.39	0.29	2, 5	2.56	0.0633	3.60
	MA7	100.42	0.41	2, 4	2.59	0.0624	3.65
	MA8	101.02	0.39	4, 5	3.19	0.0462	4.93
	MA9	101.83	0.48	1, 4, 5	4.00	0.0308	7.39
	MA10	101.84	0.48	1, 2, 4	4.01	0.0307	7.43

*Abbreviations*: *AICc* modified Akaike information criterion, *R*^*2*^ proportion of explained variation for the model, *Δ*_*i*_ difference between the AICc of the best model and the AICc for each of the other models, *Wi* Akaike weight, *ER* evidence ratio

^a^Predictor variables: 1, trophic level; 2, mean host length; 3, host species; 4, locality; 5, year

**Fig. 3:**
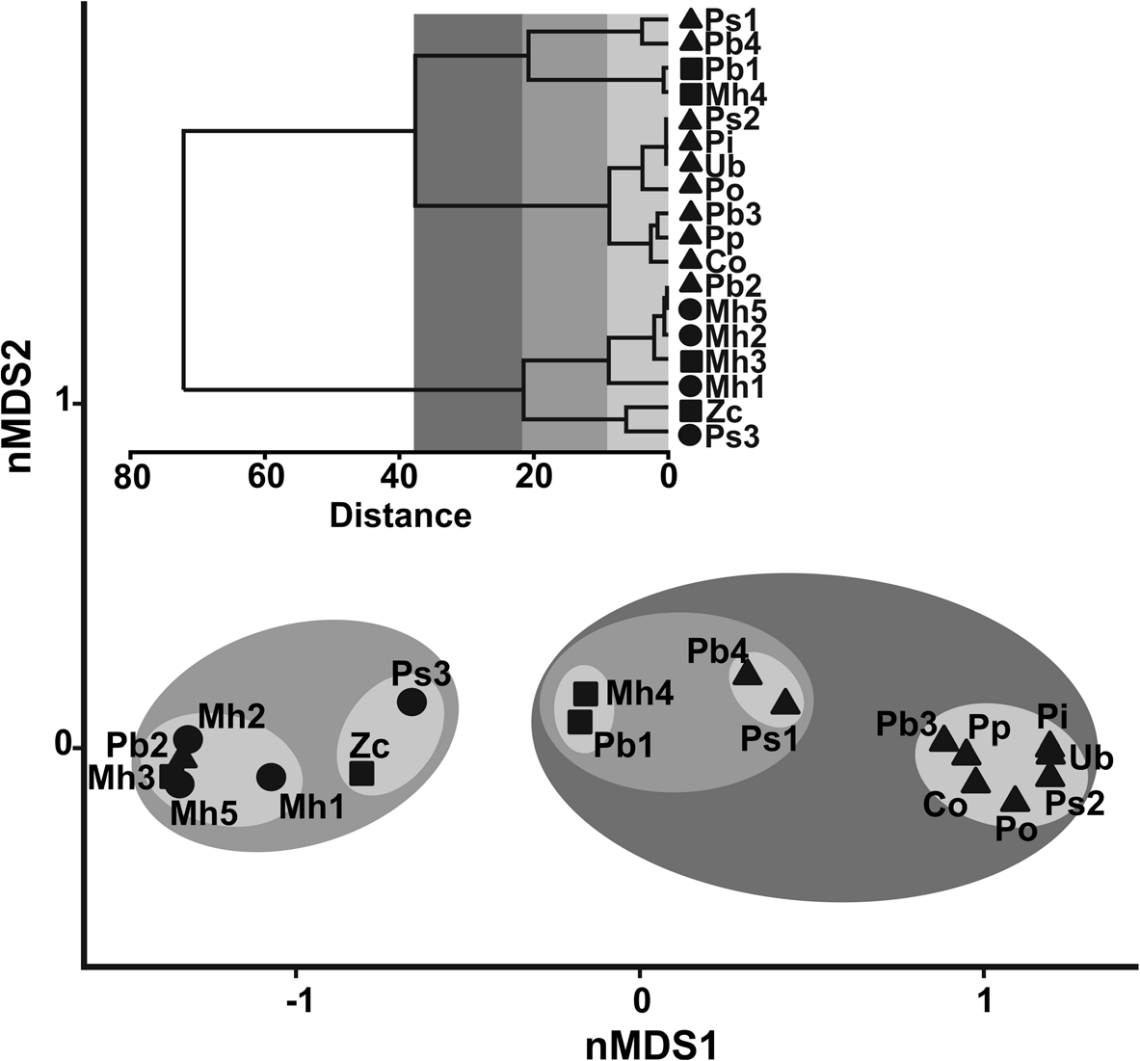
Non-metric multi-dimensional scaling plot (nMDS) and cluster analyses based on the prevalence of larval *Anisakis* in 18 samples (Euclidean distance) corresponding to 9 fish species in the southern South-West Atlantic. Results of a hierarchical agglomerative clustering are overlaid on the nMDS plot with similarity levels represented by a grey scale. Sample codes as in Table 1. Circles, Magellanic Province; triangles, Argentine Province; squares, ecotone zones


nMDS and cluster analyses on the prevalence data revealed apparent patterns of separation between samples following a zoogeographical pattern, which was substantially different from random as shown by it low stress level (0.01). Indeed, two main groups were evident (Fig. 3), one composed by most Bonaerensean samples and including samples Pb1 and Mh4 from the ecotone between Argentine and Magellanic Provinces and being more heterogeneous (branching at higher distances) than the second, which included the remaining Magellanic samples, but also the southernmost Bonaerensean Pb2 and *Z. conchifer.* A better picture of samples distribution is obtained by a three-dimensional nMDS (stress level = 0.01) (Additional file 1: Video S1).

**Additional file 1: Video S1.** Non-metric three-dimensional ordination plot based on Euclidean distances for the prevalence of larval *Anisakis* in 18 samples corresponding to 9 fish species in the southern South-West Atlantic. Red triangles, samples from the Bonaerensean District of the Argentine Province; blue triangles, samples from the Magellanic Province; green squares, samples from the ecotonal regions. Sample codes as in Table 1. (MP4 7750 kb)


## Discussion

Molecular analyses identified four *Anisakis* species in the whole sample, including *A. typica*, *A. pegreffii*, *A. berlandi* and an unidentified species, *Anisakis* sp., which seems to be conspecific with *A*. cf. *paggiae* from *Kogia sima* from off north-east Brazil [56], indicating the possible presence of a new species of *Anisakis* in the South-West Atlantic. Indeed, the K2P distance between *A. paggiae* and *Anisakis* sp. was similar to those between the sibling species of the *A. simplex* complex. Similar distances have been reported for *A. paggiae*-related species in Japanese [43], Philippine [44] and Brazilian [56] waters. Our data support the existence of an *A. paggiae* species complex as suggested by these authors. It is noteworthy that this specimen of *Anisakis* sp. is the only larva Type II so far recorded by the authors in the region, not only in the present samples, but in many other fish species previously surveyed (personal observation).

Knowledge of geographical distribution patterns of *Anisakis* spp. is scarce for many species in the genus [16]. Indeed, most species have been reported from temperate, subtropical and tropical waters between the equator and 35° north and south, being apparently more common in the boreal region [57]. The present findings increase the list of *Anisakis* species in marine fish from the West Atlantic, south to 35°S, by reporting for the first time *A. typica* and *Anisakis* sp. Together with the previously reported *A. pegreffii* and *A. berlandi*, this results in a regional richness of four species, a richness smaller than that recorded in Brazilian marine waters, where six of the nine known species of the genus [i.e. excepting *A. simplex* (*s.s.*), *A. pegreffii* and *A. berlandi*] have been recorded [56]. The record of larvae of *A. pegreffii* in a specimen of *Thunnus thynnus* from the region of Rio de Janeiro by Mattiucci et al. [58] is clearly a typing mistake (Table 3 of Mattiucci et al. [58]), which was subsequently replicated [56].

The composition of species observed in the study region reflects, furthermore, the transitional nature of its oceanographic conditions. *Anisakis typica* is known to be restricted to subtropical and tropical waters, and is the most common species in Brazilian waters [56], whereas *A. pegreffii* displays a discontinuous distribution, being known from the Mediterranean Sea, the Central East Atlantic and waters of China and Japan in the Northern Hemisphere and displaying a circumpolar distribution in the Southern Hemisphere [4, 24, 59]. *Anisakis pegreffii* is also the most reported species in the southern Argentine Sea, dominated by sub-Antarctic waters where 121 of 122 previously genetically identified worms were assigned to this species and only one to *A. berlandi* [18, 60, 61]. Therefore, *A. typica* and *A. pegreffii* can be considered as representatives of northern and southern regions of the South-West Atlantic, respectively. Despite being little represented in the examined samples, *A. berlandi* and *Anisakis* sp. also seem to have opposite geographical origins in the study region. *Anisakis berlandi* is typical from southern waters in the Southern Hemisphere [59, 61], although is also present in Pacific Canadian and Californian waters [62]. On the other hand, *Anisakis* sp. is apparently conspecific with *A*. cf. *paggiae* from *Kogia sima* from off north-east Brazil [56], a cetacean mainly distributed in deep-water habitats of tropical and temperate zones in the Central Atlantic Ocean [16]. In the marine environment, transitional zones between major water masses harbour high biodiversity, mostly due to their productivity [63], but also to an “edge effect”, as defined by Odum [64]. Indeed, ecotones contain representatives of species characteristic of adjacent communities [65], which seems to explain the specific composition of *Anisakis* in the convergence regions where masses of water supply “northern” and “southern” species. The thermal gradient produced by these marine currents in the South-West Atlantic [66] explains the dominance of *A. pegreffii* in *M. hubbsi* off Patagonia (southern waters) and of *A. typica* in *Z. conchifer*. The presence of *A. typica* in *P. pagrus* caught at similar latitudes, but in coastal waters, evidences that the influence of the Brazil Current extends to shallower areas surrounding the convergence. This sparid is associated to hard substrates and exhibits considerable site fidelity, remaining in the same patch after recruitment [67], being therefore only exposed to infective stages present in its habitat.

In addition to the environmental conditions as determinants of distribution on *Anisakis* species, their distribution patterns at large spatial scales are congruent with those of their respective final hosts [16]. *Anisakis typica* has been recorded in subtropical and tropical waters from several species of dolphins (Delphinidae), but also in the harbour porpoise, *Phocoena phocoena* (Phocoenidae) and the franciscana dolphin, *Pontoporia blainvillei* (Pontoporidae) [4, 56, 58, 68]. Most of these cetaceans are typical of warmer temperate and tropical seas [58] and consequently the distribution of the parasite can be largely determined by that of their definitive hosts. However, some of these dolphins, namely *Tursiops truncatus* and *Delphinus delphis* are also distributed in higher latitudes, reaching the colder Patagonian waters, where larvae of *A. typica* have not been recorded yet in fishes, and where the adults paraizing them have been morphologically identified as *A. simplex* (*sensu lato*) [19, 69, 70]. Similarly, *P. blainvillei* is infested by *A. typica* in Uruguayan waters, but harbours *A. simplex* (*s.l.*) in central Argentine waters [71]. On the other hand, *A. pegreffii* has been never reported in Brazilian waters. Therefore, the presence of suitable definitive hosts is a necessary but not sufficient condition to explain the geographical patterns of *Anisakis* spp., evidencing that environmental conditions play a major role in such a distribution. In agreement with most records in fish and cetacean hosts from lower latitudes [56], *A. typica* dominated the anisakid community parasitizing *Z. conchifer* in waters on the continental slope at depths between 94 and 117 m and 5° south of its known distribution limit, evidencing a higher effect of the warmer Brazil current relative to other masses of water on the local distribution of larval *Anisakis*. The influence of subtropical waters could, therefore, occur through the transport of infective stages in previous intermediate hosts or be related to the migratory behaviour of the definitive hosts or infested fish from lower latitudes. However, the possibility of *A. typica* arriving to the study zone with migratory *Z. conchifer* from northern regions seems unlikely. Although this species seems to perform ontogenetic movements to deeper waters where large fish concentrate for reproduction [72] the occurrence of latitudinal migrations has not been reported. Furthermore, the size-at-maturity of females is 311 mm (that of males is unknown) [72], which indicates that most of the examined specimens were juvenile and consequently, based on the available information, they are most probably non-migratory individuals. The presence of *A. typica* in *P. pagrus*, as mentioned above, supports the transport of infective stages by the Brazil Current as the main cause of the southern extension in the range of this parasite.

Regarding the distribution of larval *Anisakis* across fish species south of 35°S, the explanatory variables mean host length and host species had little value as drivers of the prevalence and mean abundance of parasites. The lack of relationship between host species and parasite burdens is relevant since it indicates that, given the extremely low specificity of larval *Anisakis*, fish hosts act as passive samplers of infective stages through their diets. The identity and load of larvae in fish, therefore, depend on the trophic behaviour of fish hosts, as well as on the availability of larvae in the food web, modelled by environmental conditions, in each region. Indeed, despite only fish species with a high trophic level were considered, this variable was, together with locality, the main determinant of prevalence, being also highly relevant in explaining the mean abundance of larval *Anisakis*.

Larval anisakids are long-lived in fish hosts, and can be transmitted from one paratenic host to other, persisting in the food web and being potentially available for any host, independent of its trophic level. However, trophic level determines the range of preys a predator can consume, having a direct influence on the abundance and composition of parasite communities [73, 74]. In fact, ichthyophagous fish tend to accumulate higher numbers of larval parasites because they acquire packets of helminth species that travel together in paratenic hosts along food chains [73]. Therefore, the higher their trophic level, the higher the likelihood of becoming infected and the larger the number of infective stages acquired with each individual prey [45], determining the prevalence and the mean abundance of *Anisakis* spp. regardless of the locality of capture. The year of capture also had certain relevance in determining mean abundance, indicating that some changes could have occurred in the region during recent decades; however, a reliable explanation to these variations would require additional studies, beyond the scope of the present work. Finally, and as observed for larval *Anisakis* in skates of the Argentine Sea [18], the geographical origin of the samples was the main determinant of prevalence and mean abundance of parasites. The environmental conditions in the study region are determined by the subtropical and sub-Antarctic currents flowing along the continental slope [75]. The Malvinas Current dominates adjacent shelf waters, producing a latitudinal cline of temperature which decreases southwards, whereas the effect of the warm Brazil Current is marked at the northern limit of the Argentine sea [76]. This temperature gradient, characteristic of the study area [64], provides a series of environments in which *Anisakis* larvae are differentially distributed in terms of prevalence and abundance.

DistLM analyses were carried out irrespective of the *Anisakis* species comprising the assemblages of each sample. However, nMDS analyses showed that *Z. conchifer* departed from the general zoogeographical pattern displayed by the other samples, which in turn, agreed with biogeographical schemes recognized in the South-West Atlantic [48]. Indeed, the apparent separation of samples in two groups corresponding to the Bonaerensean District of the Argentine Province and to the Magellanic Province, characterized by low and high values of prevalence, respectively, confirms the value of larval *Anisakis* as zoogeographical indicators [9, 34]. On the other hand, the two ecotonal hake samples between these two zoogeographical units (Mh3 and Mh4) clustered with Magellanic and Bonaerensean samples, respectively, indicating a higher influence of each mass of water on the populations of *Anisakis* in fish at these transitional zones.

The exception to this pattern was the assignation of the southernmost Bonaerensean sample of *P. brasiliensis* (Pb2) to the Magellanic group of samples. This sample, together with that from the ecotonal Pb1 assigned to the Bonaerensean group, displayed the higher prevalence of larval *Anisakis* for this host. Although they were considered as different stocks based on their parasite assemblages [77], Avigliano et al. [78] determined, based on otholith microchemistry, that *P. brasilisensis* from these two localities represent a single stock. In the light of the present results, migratory movements of these *P. brasiliensis* between these latitudes could not be disregarded. Finally, *Z. conchifer* from deep waters at the convergence between subtropical and sub-Antarctic currents grouped with the distant Magellanic samples as a consequence of the unexpectedly high prevalence regarding other northern samples, even some very close ones, but caught on shelf waters. Taking into account that *A. typica* was the dominant species in *Z. conchifer*, and assuming that other samples are mostly composed of *A. pegreffii*, these results could indicate that interspecific differences of populations distribution exist between these congeners. This hypothesis requires further research because no other host species with a high trophic level from a similar latitude and depth have yet been analysed. In case these differences are confirmed in other fish samples, they may be useful tools as surrogates of the identity of larvae parasitizing a given host population.

The specific identification of larval *Anisakis* in a region where they are considered among the best biological indicators for studies on zoogeography and population distribution of their hosts is undoubtedly a step forward towards the validation of the use of this methodology. Furthermore, considering the diversity of species found, new perspectives for future studies also arise, since members of *Anisakis* have proven to be excellent tools for studying host-parasite cophylogeny, as indicators of trophic web stability and indicators of habitat disturbance of marine ecosystems [4, 24].

Knowledge on the distribution of *Anisakis* spp. in the World Ocean is indispensable for future studies on the epidemiology and pathogenicity of anisakiosis, as well as on the possibility of a changing risk of this zoonosis in the time of climate change [59].

## Conclusions

The genetic identification of four species of larval *Anisakis* in fishes from the southern South-West Atlantic (three known and a probably new species related to *A. paggiae*) fill a gap in the knowledge of the global distribution of these zoonotic parasites. It was confirmed that, in the study region, larval *Anisakis* follow a clear zoogeographical pattern, being suitable indicators of tropical/subtropical and sub-Antarctic waters; the main drivers of that pattern were the host trophic level and locality of capture. This information is relevant for both human health and fishery industry, since the species in the genus exhibit differential levels of pathogenicity. The higher taxonomic resolution reached by molecular techniques represents a step forward the validation of the use of larval *Anisakis* as biological indicators for studies on host zoogeography.

## Abbreviations

AICc: Modified Akaike’s information criterion
*cox2*: Cytochrome *c* oxidase subunit 2
DistLM: Distance-based linear model
EF1 α-1: Elongation factor α-1
ER: Evidence ratio
K2P: Kimura 2-parameter model
nMDS: Non-metric multidimensional scaling
wi: Akaike weight

## References

[1] Smith JW, Wootten R (1978). *Anisakis* and anisakiasis. Adv Parasitol.

[2] Audicana MT, Ansotegui IJ, de Corres LF, Kennedy MW (2002). *Anisakis* simplex: Dangerous-dead and alive?. Trends Parasitol.

[3] Audicana MT, Kennedy MW (2008). *Anisakis simplex*: from obscure infectious worm to inducer of immune hypersensitivity. Clin Microbiol Rev.

[4] Mattiucci S, Nascetti G (2008). Advances and trends in the molecular systematics of anisakid nematodes, with implications for their evolutionary ecology and host-parasite co-evolutionary processes. Adv Parasitol.

[5] McCarthy J, Moore TA (2000). Emerging helminth zoonoses. Int J Parasitol.

[6] Timi JT (2007). Parasites as biological tags for stock discrimination in marine fish from South American Atlantic waters. J Helminthol.

[7] Lester RJG, MacKenzie K (2009). The use and abuse of parasites as stock markers for fish. Fish Res.

[8] Catalano SR, Whittington ID, Donnellan SC, Gillanders BM (2013). Parasites as biological tags to assess host population structure: guidelines, recent genetic advances and comments on a holistic approach. Int J Parasitol Parasites Wildl.

[9] Cantatore DMP, Timi JT (2015). Marine parasites as biological tags in South American Atlantic waters, current status and perspectives. Parasitology.

[10] Berland B (1961). Nematodes from some Norwegian marine fishes. Sarsia.

[11] Cipriani P, Smaldone G, Acerra V, D’Angelo L, Anastasio A, Bellisario B (2015). Genetic identification and distribution of the parasitic larvae of *Anisakis pegreffii* and *Anisakis simplex* (*s*.*s*.) in European hake *Merluccius merluccius* from the Tyrrhenian Sea and Spanish Atlantic coast: implications for food safety. Int J Food Microbiol.

[12] Mattiucci S, Abaunza P, Ramadori L, Nascetti G (2004). Genetic identification of *Anisakis* larvae in European hake from Atlantic and Mediterranean waters for stock recognition. J Fish Biol.

[13] Mattiucci S, Garcia A, Cipriani P, Santos MN, Nascetti G, Cimmaruta R (2014). Metazoan parasite infection in the swordfish, *Xiphias gladius*, from the Mediterranean Sea and comparison with Atlantic populations: implications for its stock characterization. Parasite.

[14] Mattiucci S, Cimmaruta R, Cipriani P, Abaunza P, Bellisario B, Nascetti G (2015). Integrating *Anisakis* spp. parasites data and host genetic structure in the frame of a holistic approach for stock identification of selected Mediterranean Sea fish species. Parasitology.

[15] Abaunza P, Murta AG, Campbell N, Cimmaruta R, Comesana AS, Dahle G (2008). Stock identity of horse mackerel (*Trachurus trachurus*) in the Northeast Atlantic and Mediterranean Sea: integrating the results from different stock identification approaches. Fish Res.

[16] Kuhn T, García-Márquez J, Klimpel S (2011). Adaptive radiation within marine anisakid nematodes: a zoogeographical modelling of cosmopolitan, zoonotic parasites. PLoS One.

[17] Gómez-Mateos M, Valero A, Morales-Yuste M, Martín-Sánchez J (2016). Molecular epidemiology and risk factors for *Anisakis simplex s.l.* infection in blue whiting (*Micromesistius poutassou*) in a confluence zone of the Atlantic and Mediterranean: differences between *A. simplex s.s.* and *A. pegreffii*. Int J Food Microbiol.

[18] Irigoitia MM, Braicovich PE, Lanfranchi AL, Farber MD, Timi JT (2018). Distribution of anisakid nematodes parasitizing rajiform skates under commercial exploitation in the Southwestern Atlantic. Int J Food Microbiol.

[19] Hernández-Orts JS, Paso Viola MN, García NA, Crespo EA, González R, García-Varela M, Kuchta R (2015). A checklist of the helminth parasites of marine mammals from Argentina. Zootaxa.

[20] Berón-Vera B, Crespo EA, Raga JA (2008). Parasites in stranded cetaceans of Patagonia. J Parasitol.

[21] Luque JL, Muniz-Pereira LC, Siciliano S, Siqueira LR, Oliveira MS, Vieira FM (2010). Checklist of helminth parasites of cetaceans from Brazil. Zootaxa.

[22] Fonseca MCG, Knoff M, Felizardo NN, Di Azevedo MIN, Torres EJL, Gomes DC (2016). Integrative taxonomy of Anisakidae and Raphidascarididae (Nematoda) in *Paralichthys patagonicus* and *Xystreurys rasile* (Pisces: Teleostei) from Brazil. Int J Food Microbiol.

[23] Di Azevedo MIN, Iñiguez AM (2018). Nematode parasites of commercially important fish from the southeast coast of Brazil: morphological and genetic insight. Int J Food Microbiol.

[24] Mattiucci S, Paoletti M, Cipriani P, Webb SC, Timi JT, Nascetti G, Klimpel S, Kuhn T, Mehlhorn H (2017). Inventorying biodiversity of anisakid nematodes from the Austral Region: a hotspot of genetic diversity?. Biodiversity and Evolution of Parasitic Life in the Southern Ocean. Parasitology Research Monographs.

[25] Piola AR, Martínez Avellaneda N, Guerrero RA, Jardón FP, Palma ED, Romero SI (2010). Malvinas-slope water intrusions on the northern Patagonia continental shelf. Ocean Sci.

[26] Moreno IB, Zerbini AN, Danilewicz D, de Oliveira Santos MC, Simões-Lopes PC, Lailson-Brito J, Azevedo AF (2005). Distribution and habitat characteristics of dolphins of the genus *Stenella* (Cetacea: Delphinidae) in the southwest Atlantic Ocean. Mar Ecol Prog Ser.

[27] Mandiola MA, Giardino GV, Bastida J, Rodríguez DH, Bastida RO (2015). Marine mammal occurrence in deep waters of the Brazil-Malvinas Confluence off Argentina during summer. Mastozool Neotrop.

[28] Di Tullio JC, Gandra TBR, Zerbini AN, Secchi ER (2016). Diversity and distribution patterns of cetaceans in the subtropical southwestern Atlantic outer continental shelf and slope. PLoS One.

[29] Lanfranchi AL, Braicovich PE, Cantatore DMP, Alarcos AJ, Luque JL, Timi JT (2016). Ecotonal marine regions - ecotonal parasite communities: helminth assemblages in the convergence of masses of water in the southwestern Atlantic. Int J Parasitol.

[30] Haimovici M, Martins AS, Figueiredo JL, Vieira PC (1994). Demersal bony fish of the outer shelf and upper slope of the southern Brazil Subtropical Convergence ecosystem. Mar Ecol Prog Ser.

[31] Quéro JC, Du Buit MH, Vayne JJ (1998). Les observations de poisons tropicaux et le réchauffement des eaux dans l’Atlantique européen. Oceanol Acta.

[32] Ragonese S, Giusto GB (2007). *Zenopsis conchifera* (Lowe, 1852) (Pisces, Actinopterygii, Zeidae): a new alien fish in the Mediterranean Sea. J Fish Biol.

[33] Pereira AN, Pantoja C, Luque JL, Timi JT (2014). Parasites of *Urophycis brasiliensis* (Gadiformes: Phycidae) as indicators of marine ecoregions in coastal areas of the South American Atlantic with the assessment of their stocks. Parasitol Res.

[34] Braicovich PE, Pantoja C, Pereira AN, Luque JL, Timi JT (2017). Parasites of the Brazilian flathead *Percophis brasiliensis* reflect West Atlantic biogeograhic regions. Parasitology.

[35] dos Reis Sardella CJ, Luque JL (2016). Diagnóstico morfológico e molecular de larvas de *Anisakis typica* e *Anisakis brevispiculata* em peixes do litoral do Rio de Janeiro. Braz J Vet Med.

[36] Bush AO, Lafferty KD, Lotz JM, Shostak AW (1997). Parasitology meets ecology on its own terms: Margolis et al*.* revisited. J Parasitol.

[37] Nadler SA, Hudspeth DSS (2000). Phylogeny of the Ascaridoidea (Nematoda: Ascaridida) based on three genes and morphology: hypothesis of structural and sequence evolution. J Parasitol.

[38] Mattiucci S, Acerra V, Paoletti M, Cipriani P, Levsen A, Webb SC (2016). No more time to stay ‘single’ in the detection of *Anisakis pegreffii*, *A. simplex* (*s.s.*) and hybridization events between them: a multi-marker nuclear genotyping approach. Parasitology.

[39] Filatov DA (2002). ProSeq: a software for preparation and evolutionary analysis of DNA sequence data sets. Mol Ecol Notes.

[40] Altschul FS, Gish W, Miller W, Myers EW, Lipman DJ (1990). Basic local alignment search tool. J Mol Biol.

[41] Thompson JD, Higgins DG, Gibson TJ (1994). CLUSTAL W: improving the sensitivity of progressive multiple sequence alignment through sequence weighting, position-specific gap penalties and weight matrix choice. Nucleic Acids Res.

[42] Kumar S, Stecher G, Tamura K (2016). MEGA7: Molecular Evolutionary Genetics Analysis version 7.0 for bigger datasets. Mol Biol Evol.

[43] Quiazon K, Yoshinaga T, Santos M, Ogawa K (2009). Identification of larval *Anisakis* spp. (Nematoda: Anisakidae) in Alaska pollock (*Theragra chalcogramma*) in northern Japan using morphological and molecular markers. J Parasitol.

[44] Quiazon K, Santos M, Yoshinaga T (2013). *Anisakis* species (Nematoda: Anisakidae) of dwarf sperm whale *Kogia sima* (Owen, 1866) stranded off the Pacific coast of southern Philippine archipelago. Vet Parasitol.

[45] Timi JT, Rossin MA, Alarcos AJ, Braicovich PE, Cantatore DMP, Lanfranchi AL (2011). Fish trophic level and the similarity of larval parasite assemblages. Int J Parasitol.

[46] Froese R, Pauly D (2018). Fish Base. World Wide Web Electronic Publication.

[47] Cousseau MB, Perrotta RG (2013). Peces Marinos de Argentina: Biología, Distribución, Pesca.

[48] Menni RC, Jaureguizar AJ, Stehmann MFW, Lucifora LO (2010). Marine biodiversity at the community level: zoogeography of sharks, skates, rays and chimaeras in the southwestern Atlantic. Biodivers Conserv.

[49] Anderson MJ, Gorley RN, Clarke KR (2008). PERMANOVA+ for PRIMER: Guide to Software and Statistical Methods.

[50] Timi JT, Lanfranchi AL (2013). Ontogenetic changes in heterogeneity of parasite communities of fish: disentangling the relative role of compositional *versus* abundance variability. Parasitology.

[51] Braicovich PE, Ieno EN, Sáez M, Despos J, Timi JT (2016). Assessing the role of host traits as drivers of the abundance of long-lived parasites in fish stock assessment studies. J Fish Biol.

[52] Symonds MRE, Moussalli A (2011). A brief guide to model selection, multimodel inference and model averaging in behavioural ecology using Akaike’s information criterion. Behav Ecol Sociobiol.

[53] Burnham KP, Anderson DR (2002). Model Selection and Multimodel Inference.

[54] Richards SA (2005). Testing ecological theory using the information theoretic approach: examples and cautionary results. Ecology.

[55] Clarke KR, Gorley RN (2015). PRIMER v7: User Manual/Tutorial.

[56] Di Azevedo MIN, Carvalho VL, Iñiguez AM (2017). Integrative taxonomy of anisakid nematodes in stranded cetaceans from Brazilian waters: an update on parasite’s hosts and geographical records. Parasitol Res.

[57] Klimpel S, Kuhn T, Busch MW, Horst K, Palm HW (2011). Deep water life-cycle of *Anisakis paggiae* (Nematoda: Anisakidae) in the Irminger Sea indicates kogiid whale distribution in North Atlantic waters. Polar Biol.

[58] Mattiucci S, Paggi L, Nascetti G, Santos CP, Costa G, Di Beneditto AP (2002). Genetic markers in the study of *Anisakis typica* (Diesing, 1860): larval identification and genetic relationships with other species of *Anisakis* Dujardin, 1845 (Nematoda: Anisakidae). Syst Parasitol.

[59] Klimpel Sven, Palm Harry W. (2011). Anisakid Nematode (Ascaridoidea) Life Cycles and Distribution: Increasing Zoonotic Potential in the Time of Climate Change?. Progress in Parasitology.

[60] Mattiucci S., Nascetti G., Cianchi R., Paggi L., Arduino P., Margolis L., Brattey J., Webb S., D'Amelio S., Orecchia P., Bullini L. (1997). Genetic and Ecological Data on the Anisakis simplex Complex, with Evidence for a New Species (Nematoda, Ascaridoidea, Anisakidae). The Journal of Parasitology.

[61] Mattiucci S, Nascetti G (2007). Genetic diversity and infection levels of anisakid nematodes parasitic in fish and marine mammals from Boreal and Austral hemispheres. Vet Parasitol.

[62] Klimpel S, Busch M, Khun T, Rohde A, Palm HW. The *Anisakis simplex* complex off the South Shetland Islands (Antarctica): endemic populations *versus *introduction through migratory hosts. Mar Ecol Prog Ser. 2010;34:899–906.

[63] Scales KL, Miller PI, Hawkes LA, Ingram SN, Sims DW, Votier SC (2014). On the front line: frontal zones as priority at-sea conservation areas for mobile marine vertebrates. J Appl Ecol.

[64] Odum EP (1959). Fundamentals of Ecology.

[65] Baker J, French K, Whelan RJ (2002). The edge effect and ecotonal species: bird communities across a natural edge in southeastern Australia. Ecology.

[66] Hoffmann J, Núñez M, Piccolo M, Boschi EE (1997). Características climáticas del océano Atlántico sudoccidental. El Mar Argentino y sus Recursos Pesqueros Tomo I. Antecedentes históricos de las exploraciones en el mar y las características ambientales.

[67] De Vries AD. The life history, reproductive ecology and demography of the red porgy, Pagrus pagrus, in the northeastern Gulf of Mexico. PhD Dissertation. Tallahassee: Florida State University; 2006.

[68] Iñiguez AM, Carvalho VL, Motta MR, Pinheiro DC, Vicente AC (2011). Genetic analysis of *Anisakis typica* (Nematoda: Anisakidae) from cetaceans of the northeast coast of Brazil: new data on its definitive hosts. Vet Parasitol.

[69] Berón-Vera B, Crespo EA, Raga JA, Fernández M (2007). Parasites communities of common dolphins (*Delphinus delphis*) from Patagonia: the relation with host distribution and diet and comparison with sympatric host. J Parasitol.

[70] Romero MA, Fernández M, Dans SL, García NA, González R, Crespo EA (2014). Gastrointestinal parasites of bottlenose dolphins *Tursiops truncatus* from the extreme south western Atlantic, with notes on diet composition. Dis Aquat Org.

[71] Aznar FJ, Raga JA, Corcuera J, Monzón F (1995). Helminths as biological tags for franciscana (*Pontoporia blainvillei*) (Cetacea, Pontoporiidae) in Argentinian and Uruguayan waters. Mammalia.

[72] Martins RS, Schwingel PR (2012). Biological aspects of the sailfin dory *Zenopsis conchifer* (Lowe, 1852) caught by deep-sea trawling fishery off southern Brazil. Braz J Oceanogr.

[73] Marcogliese DJ (2002). Food webs and the transmission of parasites to marine fish. Parasitology.

[74] Chen H-W, Liu W-C, Davis AJ, Jordan F, Hwang MJ, Shao K-T (2008). Network position of hosts in food webs and their parasite diversity. Oikos.

[75] Piola AR, Rivas AL, Boschi EE (1997). Corrientes en la plataforma continental. El Mar Argentino y Sus Recursos Pesqueros Tomo I. Antecedentes históricos de las exploraciones en el mar y las características ambientales.

[76] Guerrero RA, Piola AR, Boschi EE (1997). Masas de agua en la plataforma continental. El Mar Argentino y sus Recursos Pesqueros Tomo I. Antecedentes históricos de las exploraciones en el mar y las características ambientales.

[77] Braicovich PE, Timi JT (2008). Parasites as biological tags for stock discrimination of the Brazilian flathead *Percophis brasiliensis* in the south-west Atlantic. J Fish Biol.

[78] Avigliano Esteban, Saez Margarita B., Rico Rita, Volpedo Alejandra V. (2015). Use of otolith strontium:calcium and zinc:calcium ratios as an indicator of the habitat of Percophis brasiliensis Quoy & Gaimard, 1825 in the southwestern Atlantic Ocean. Neotropical Ichthyology.

[79] Sardella NH, Timi JT (2004). Parasites of Argentine hake in the Argentine Sea: population and infracommunity structure as evidence for host stock discrimination. J Fish Biol.

[80] Alarcos AJ, Timi JT (2012). Parasite communities in three sympatric flounder species (Pleuronectiformes: Paralichthyidae). Similar ecological filters driving toward repeatable assemblages. Parasitol Res.

[81] Alarcos AJ, Etchegoin JA (2010). Parasite assemblages of estuarine-dependent marine fishes from Mar Chiquita coastal lagoon (Buenos Aires Province, Argentina). Parasitol Res.

[82] Timi JT, Lanfranchi AL (2009). The metazoan parasite communities of the Argentinean sandperch *Pseudopercis semifasciata* (Pisces: Perciformes) and their use to elucidate the stock structure of the host. Parasitology.

